# Role of Adjuvant Chemotherapy in Stage I Pure Ovarian Immature Teratoma: A Systematic Review and Meta-Analysis

**DOI:** 10.3390/cancers15061741

**Published:** 2023-03-13

**Authors:** Sijian Li, Yuelin Wang, Xinyue Zhang, Tianyu Zhang, Min Yin, Jiaxin Yang

**Affiliations:** 1 National Clinical Research Center for Obstetric and Gynecologic Diseases, Department of Obstetrics and Gynecology, Peking Union Medical College Hospital, Chinese Academy of Medical Sciences, Peking Union Medical College, Beijing 100730, China; 2 Department of Ophthalmology, Peking Union Medical College Hospital, Chinese Academy of Medical Sciences, Peking Union Medical College, Beijing 100730, China

**Keywords:** malignant germ cell tumors, ovarian immature teratoma, active surveillance, adjuvant chemotherapy, survival outcomes

## Abstract

**Simple Summary:**

A systematic review and meta-analysis was performed to determine the role of adjuvant chemotherapy in FIGO stage IA G2-3 and stage IB-IC pure ovarian immature teratoma (POIT) and 15 studies with 707 patients were enrolled. Compared with surveillance, adjuvant chemotherapy significantly decreased the mortality rate (RR 0.31, 95% CI 0.11–0.88, *p* = 0.03), but not recurrence (*p* = 0.37), in the overall cohort. Subgroup analysis showed no statistical difference in the recurrence rate and mortality rate between patients who received adjuvant chemotherapy and surveillance in pediatric POIT, stage IA G2-3 POIT, stage IB-IC POIT, and stage IA-IC G3 POIT. Patients who received adjuvant chemotherapy appeared to have a lower risk of both recurrence (RR 0.17, 95% CI 0.03–0.83, *p* = 0.03) and death (*p* = 0.05) in adult POIT. Surveillance in stage I POIT over IA G1 should be applied cautiously, especially in adult patients.

**Abstract:**

To determine the role of adjuvant chemotherapy in stage IA G2-3 and stage IB-IC pure ovarian immature teratoma (POIT), we performed a systematic review and meta-analysis by searching PubMed, Embase, Cochrane library, Web of Science, and ClinicalTrials.gov. Randomized controlled trials or cohort studies on stage IA G2-G3 or stage IB-IC POIT between 1 January 1970 and 15 December 2022 were enrolled. The recurrence rate and mortality rate were the primary outcomes, and subgroup analysis based on the tumor stage and grade was also conducted. In total, 15 studies with 707 patients were included. Compared with surveillance, adjuvant chemotherapy significantly decreased the mortality rate (RR 0.31, 95% CI 0.11–0.88, *p* = 0.03), but not recurrence (RR 0.74, 95% CI 0.39–1.42, *p* = 0.37), in the overall population. Subgroup analysis showed no statistical difference in the recurrence rate and mortality rate between patients who received adjuvant chemotherapy and surveillance in pediatric POIT, stage IA G2-3 POIT, stage IB-IC POIT, and stage IA-IC G3 POIT (all with *p* > 0.05). However, patients who underwent adjuvant chemotherapy appeared to have a lower risk of both recurrence (RR 0.17, 95% CI 0.03–0.83, *p* = 0.03) and death (RR 0.04, 95% CI 0.00–1.00, *p* = 0.05) in adult POIT. Adjuvant chemotherapy significantly decreased the mortality rate in patients with stage I POIT and lowered the risk of recurrence in the adult subgroup. Surveillance administered in stage I POIT over IA G1 should be cautious, especially in adult patients.

## 1. Introduction

Pure ovarian immature teratoma (POIT) is one of the most common subtypes of malignant ovarian germ cell tumors (MOGCTs) that contain tissue derived from three germ layers and immature neural components, comprising approximately one-third of cases [1,2]. POIT is staged according to the International Federation of Gynecology and Obstetrics (FIGO) [3] and graded per the criteria modified by Scully and Robboy [4]. POIT predominantly affects young patients and presents at an early stage, so fertility-sparing surgery with optimal surgical staging is the most commonly applied surgical treatment [5]. For patients with stage IA G1 POIT, unilateral salpingo-oophorectomy with comprehensive surgical staging without adjuvant chemotherapy is recommended by both the ESMO and NCCN guidelines. However, whether surveillance or adjuvant chemotherapy in stage I POIT patients, except IA G1, remains controversial [5,6].

The ESMO guidelines suggest that POIT at the IB-IC stages should receive 3–4 cycles of bleomycin, etoposide, and cisplatin (BEP) chemotherapy after surgery, while active surveillance could be preserved in stage IA G2-G3 POIT with negative postoperative tumor markers after properly staged patients [5]. However, according to the NCCN guidelines, surveillance after surgery is only recommended for stage IA-IC G1 POIT [6]. Additionally, in pediatric POIT, surveillance in stage I of any grade is recommended and has been proven to be safe as well as to reduce chemotherapy-related adverse events [7,8,9]. Recently, a series of studies proposed that active surveillance may be acceptable in stage IA-IC POIT of any grade, regardless of whether patients are pediatric or adult [10,11,12,13,14,15]. Nonetheless, most of these studies have evaluated pediatric and adult patients together or included patients with different pathological subtypes of MOGCTs [12,13,15]. Furthermore, due to the relatively small sample size and retrospective nature, the evidence favoring surveillance or adjuvant chemotherapy in each corresponding tumor stage (IA, IB, IC) and grade (G1, G2, G3) is insufficient [10,11]. The significance of postoperative adjuvant chemotherapy in patients with stage I POIT, except IA G1, still needs to be explored.

To further investigate the role of adjuvant chemotherapy in patients with stage IA G2-3 and stage IB-IC POIT in any grade, we conducted a systematic review and meta-analysis that integrated the published research. The impact of adjuvant chemotherapy on recurrence and death was evaluated, and subgroup analyses according to age at presentation, tumor stage, and grade were also performed.

## 2. Materials and Methods

This meta-analysis was conducted following the Preferred Reporting Items for Systematic Reviews and Meta-Analysis (Text S1). To ensure transparency, reliability, and novelty, we registered the protocol for this study in the Prospective Register of Systematic Reviews (ID: CRD42023387224).

### 2.1. Data Sources, Search Strategy, and Selection Criteria

To collect all available data published, we systematically searched PubMed, EMBASE, Cochrane Library, Web of Science, and ClinicalTrials.gov from 1 January 1970 to 15 December 2022. Relevant systematic reviews, conference proceedings, international trial registers, and reference mining of relevant publications were also reviewed to identify additional literature. The keywords for the literature search were as follows: “ovarian immature teratoma”, “ovarian malignant teratoma”, “malignant ovarian germ cell tumors”, “MOGCT”, and “adjuvant chemotherapy” (Text S2).

The inclusion criteria were listed as follows:

(1) Patients with stage IA G2-G3 and/or IB-IC POIT of any grade confirmed by pathology;
(2) Randomized controlled trials (RCTs) or prospective, retrospective cohort studies that included POIT treated with surgery alone and surgery with adjuvant chemotherapy;
(3) Studies that exactly reported outcomes (death or recurrence) of POIT based on intervention (surgery or surgery with adjuvant chemotherapy), corresponding stage (FIGO stage IA, IB, or IC), and/or WHO grade (G1, G2, or G3).

Studies that met the following criteria were excluded:

(1) POIT of other stages/grades or topics, or studies that enrolled less than 10 cases of POIT that met the inclusion criteria;
(2) MOGCTs of other pathology subtypes;
(3) Patients reported in case reports, letters, personal opinions, conference abstracts, and non-English literature;
(4) Studies with ambiguous clinical outcomes or unclear tumor stage/grade.

The titles and abstracts of the selected literature were screened by two authors (SJ L, YL W) before the assessment of full texts to determine eligibility. All of the included studies were double-checked online to ensure the inclusion of the most recent data. If two authors disagreed, a third researcher (XY Z) participated in the discussion.

### 2.2. Data Extraction and Quality Assessment

Two investigators (SJ L and YL W) independently extracted the data according to the Preferred Reporting Items for Systematic Reviews and Meta-Analysis [16] process, and any discrepancies were solved by discussions with the other three authors (XY Z, MY, and TY Z). Detailed information, particularly sample size, tumor stage, tumor grade, intervention (surgery alone, namely surveillance, or adjuvant chemotherapy), and the events of results (recurrence or death) were extracted from each article. Patients with unclear characteristics, treatment, outcomes, etc., were omitted, even in the included studies. The quality of the included RCTs was assessed using the Cochrane collaborative’s risk for bias-assessment tool [17]. The quality of the included non-RCTs was assessed using the Newcastle–Ottawa Scale. We solved any potential disagreements through discussion.

### 2.3. Outcomes and Subgroup Setting

In this study, the primary outcome was the recurrence rate and mortality rate in patients with stage I POIT (overall population) who received adjuvant chemotherapy or not. Importantly, we defined recurrence as the pathology-confirmed presence of immature components, as mature teratoma would not be considered as recurrence. The secondary outcomes were the recurrence rate and mortality rate in patients who received adjuvant chemotherapy or not in each subgroup. The subgroups were set as IA G2-G3 POIT, stage I G3 POIT, IB/IC POIT, IB/IC G2-G3 POIT, pediatric (<18 years), and adult (≥18 years) POIT.

### 2.4. Statistical Analysis

The recurrence rate and death rate of the overall cohort were the primary outcomes; the secondary outcomes were the recurrence rate and death rate according to age (pediatric or adult), tumor stage (IA, IB, IC), and tumor grade (WHO G1, G2, G3). RevMan 5.4 (Cochrane Review software) was used to perform statistical analysis including pooling the data and producing the forest plots. Pooled risk ratios (RR) and 95% CI were used for dichotomous outcomes. We used the Mantel–Haenszel (M-H) method to combine the summary statistics and assessed the statistical heterogeneity using the I^2^ method with the χ^2^ test to calculate the *p* values, and a two-tailed *p*-value < 0.05 was considered significant. Heterogeneity was evaluated using the I^2^ statistic. A fixed-effects model was applied to perform meta-analysis if I^2^ was less than 50%; otherwise, a random-effects model was used. Potential publication bias was assessed by the Egger test, with *p* > 0.05 indicating negative publication bias using Stata (SE v12, StataCorp, College Station, TX, USA).

## 3. Results

### 3.1. Systematic Review and Characteristics of the Included Studies

**Figure 1:**
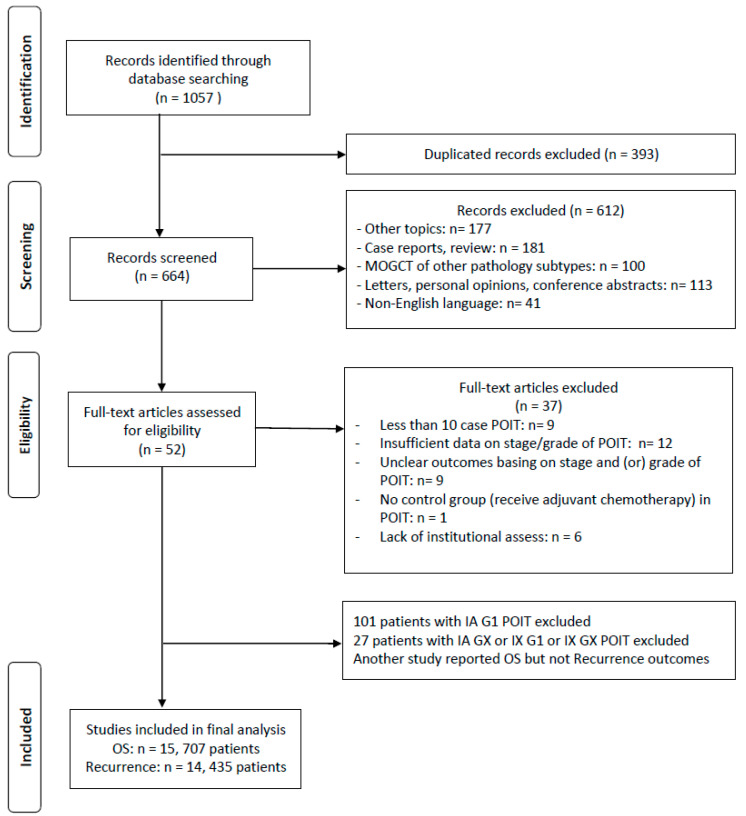
The PRISMA flow diagram showing the inclusions detailed in this study.

We initially identified a total of 1057 studies from the databases and additional records during the preliminary literature search. After eliminating the duplicates and screening the titles and abstracts, 52 studies were selected for full-text assessment (Figure 1). Eventually, we included 15 studies [10,11,12,13,15,18,19,20,21,22,23,24,25,26] of 707 patients in our meta-analysis (Table 1), of which 14 studies consisting of 435 patients reported outcomes for both the recurrence and mortality rate [10,11,12,13,15,18,19,20,21,22,23,25,26]. Importantly, we excluded 101 patients with stage IA G1 POIT and 27 patients with IA GX, IX G1, or IX GX diseases within these included studies. Among the included manuscripts, 14 of these studies were retrospective single-center or multicenter cohort studies, and the other one was a prospective cohort study. Literature quality evaluation revealed the moderate-to-high quality of the included cohort studies. The literature quality evaluation can be found in Table S1. The major chemotherapy regimens were BEP or bleomycin, vincristine, and cisplatin (BVP), and some patients were treated with etoposide and cisplatin (EP). Meanwhile, some studies did not clearly state the regimens or dose (Table S2).

### 3.2. Primary Outcomes

**Figure 2:**
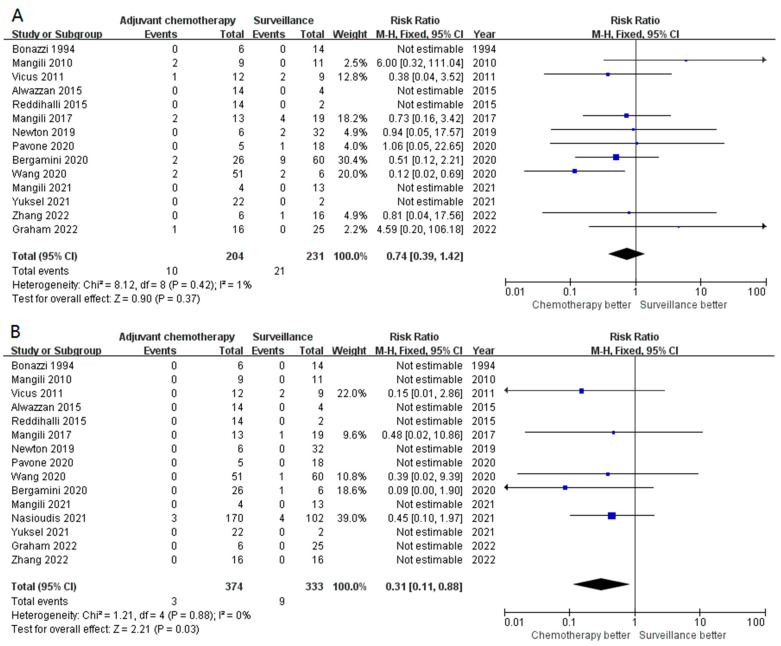
The forest plot demonstrates the impact of adjuvant chemotherapy in stage I POIT in the overall population. (**A**) Impact of adjuvant chemotherapy in disease recurrence; (**B**) surveillance significantly improved the DSS.

Fourteen studies reported the recurrence rate in stage I POIT patients who received adjuvant chemotherapy or not. Overall, 9.1% (21/231) and 4.9% (10/204) of the patients experienced the recurrence of those who received surveillance and adjuvant chemotherapy after surgical treatment, respectively. However, adjuvant chemotherapy did not significantly lower the possibility of recurrence compared with surveillance (RR 0.74, 95% CI 0.39–1.42, *p* = 0.37) (Figure 2A). No obvious heterogeneity was observed among these studies (I^2^ = 1%, *p* = 0.42). The disease-specific survival (DSS) was excellent in patients with stage I POIT and the pooled mortality rate was 1.70% (12/707), all of which were attributed to the tumor. Interestingly, compared with surveillance, postoperative adjuvant chemotherapy significantly improved the DSS (RR 0.31, 95% CI 0.11–0.88, *p* = 0.03) (Figure 2B). Similarly, the heterogeneity was weak, with an I^2^ of 0% (*p* = 0.88). The Egger’s test indicated a negative publication bias (*p* = 0.496).

Due to the insufficient survival data after we excluded patients with IA G1, IX G1, and IX GX, the synthetic 5-year recurrence-free survival (RFS) and 5-year overall survival (OS)/DSS rates were unavailable.

### 3.3. Secondary Outcomes (Subgroup Analysis)

Subgroup analysis was performed to further investigate whether the age of patients, tumor stage, and tumor grade may alter the significance of adjuvant chemotherapy in POIT. Due to the limited cases of stage IB POIT, we classified stage IB-IC as one subgroup. Similarly, patients with IA-IC G3 POIT were classed as one subgroup (stage I G3) rather than as IA G3 or IB-IC G3 subgroups. However, the 5-year RFS and 5-year OS/DSS rates were again not applicable to retrieve from the included literature.

Forty-three patients aged younger than eighteen years old met the inclusion criteria in our manuscript that were assigned to the pediatric subgroup. Two patients relapsed, one each in the surveillance group and adjuvant chemotherapy group. In pediatric POIT, we found no statistical difference in recurrence (RR 2.33, 95% CI 0.29–18.74, *p* = 0.43, Figure S1A) and DSS (no event in two groups, Figure S1B) in patients who underwent adjuvant chemotherapy and surveillance. A total of 106 POIT patients ≥18 years old identified in four included studies were subjected to the adult subgroup. However, the forest plot showed that surgery with postoperative adjuvant chemotherapy had a significantly lower risk of recurrence (RR 0.17, 95% CI 0.03–0.83, *p* = 0.03) and death (RR 0.04, 95% CI 0.00–1.00, *p* = 0.05) (Figure S1C,D) compared with surgery followed by surveillance. The detailed stages and grades for patients in these two subgroups can be found in Table S3.

**Figure 3:**
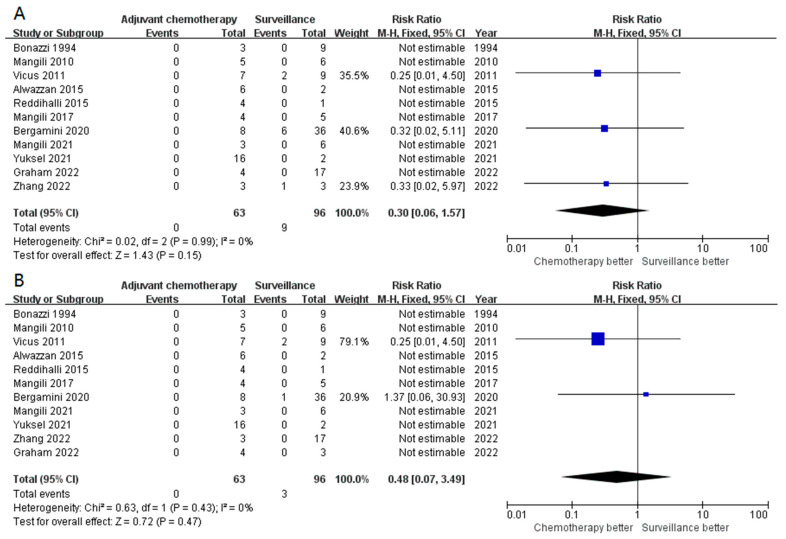
The forest plot revealed no significant difference between the stage IA G2-G3 POIT patients who received adjuvant chemotherapy or surveillance in recurrence (**A**) or mortality (**B**).

**Figure 4:**
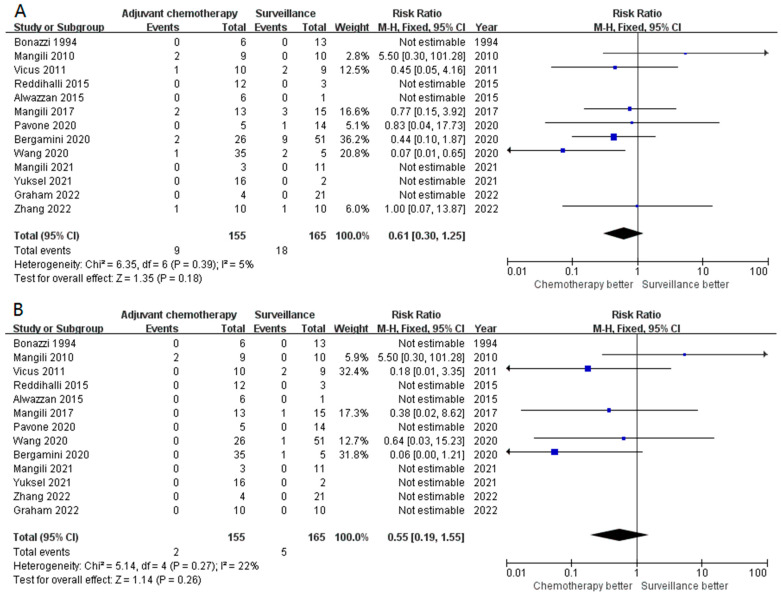
The risk of recurrence (**A**) and mortality (**B**) did not differ between patients with stage I G2-G3 POIT who underwent adjuvant chemotherapy and surveillance.

**Figure 5:**
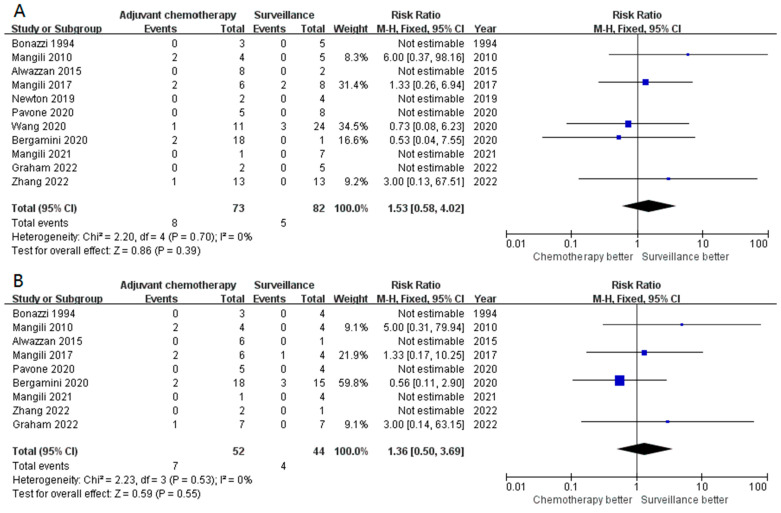
Compared with surveillance, adjuvant chemotherapy did not significantly decrease the risk of recurrence in stage IB-IC POIT (**A**) and stage IB-IC G2-G3 POIT (**B**).

We further assessed the impact of adjuvant chemotherapy in other subgroups. In 155 patients with stage IA G2-3 disease, adjuvant chemotherapy did not improve the survival outcomes in terms of both recurrences (RR 0.30, 95% CI 0.06–1.57, *p* = 0.15) and mortality (RR 0.48, 95% CI 0.07–3.49, *p* = 0.47) (Figure 3A,B). Similarly, adjuvant chemotherapy seemed to not be superior to surveillance in both recurrence (*p* = 0.18, Figure 4A) and DSS (*p* = 0.26, Figure 4B) in patients with stage I G2-3 POIT. Furthermore, adjuvant chemotherapy appeared to add no survival benefit, even in the stage I G3 subgroup (*p* = 0.14 for recurrence and *p* = 0.16 for DSS, Figure S2A,B). A total of 155 and 96 patients were classified as stage IB-IC of any grade and stage IB-IC of G2-3 disease, respectively. The forest plot again demonstrated comparable results in both recurrence (*p* = 0.39, Figure 5A) and death (*p* = 0.21, Figure S3A) in stage IB-IC POIT of any grade. Strikingly, in patients with stage IB-IC POIT of G2-G3, neither the recurrence rate (*p* = 0.55, Figure 5B) nor the mortality rate (no event, Figure S3B) were decreased in those who received adjuvant chemotherapy when compared with those underwent surveillance.

## 4. Discussion

Our study presents the first systematic review and meta-analysis of patients with stage I POIT, focusing on the role of adjuvant chemotherapy. Based on the largest cohort yet studied, we found that compared with surveillance, adjuvant chemotherapy significantly decreased the mortality rate in patients with stage I POIT. The subsequent subgroup analysis revealed that adjuvant chemotherapy was associated with a lower risk of recurrence and death in adult patients, but it did not reduce the chance of recurrence and death in pediatric POIT, IA G2-3 POIT, and IB-IC POIT.

Chemotherapy de-escalation for stage I MOGCTs has raised great awareness in recent years due to chemotherapy-induced toxicities including the risk of kidney or hearing impairment, secondary cancers, peripheral neuropathy, and irreversible pulmonary fibrosis [27,28]. However, unlike testicular germ cell tumors (TGCT), platinum-based chemotherapy, especially BEP after comprehensive staging surgery, has always been the standard treatment of MOGCTs, except for stage IA G1 POIT and stage I dysgerminoma [5,6]. Researchers have evaluated the safety of extending surveillance to stage IA-IC POIT of any grade [10,11,12,13,25]. Nonetheless, most of these studies enrolled various histological types of MOGCTs, and the division of subgroups between the FIGO stage and WHO grade differed, weakening the strength to support active surveillance in all stage I POIT patients. Moreover, one systematic review and meta-analysis also investigated the significance of chemotherapy in adult MOGCTs [29]. However, it only included 32 patients with MOGCTs, of which 13 patients were diagnosed as POIT, making it impractical to draw any conclusions. Our current research further addressed the role of adjuvant chemotherapy in stage I POIT, regardless of age, tumor stage, and tumor grade.

In our study, patients with stage I POIT who received adjuvant chemotherapy had a significantly better DSS compared with those who underwent surveillance. This was inconsistent with previous studies [10,11] and may be partially attributed to the different inclusion criteria and sample sizes, as those who reported comparable DSS outcomes in patients administered surveillance or adjuvant chemotherapy mostly [12,13,15] included IA G1 POIT in their analysis, while only three studies [10,11,24] included more than 50 patients that met our inclusion criteria. In 127 patients, we excluded in those included studies where only one recurrence each was found in two groups and none of them died, inevitably altering the true impact of adjuvant chemotherapy in the survival outcomes. Furthermore, we observed that all of the five studies that reported death tended to favor adjuvant chemotherapy in the forest plot, indicating that adjuvant chemotherapy indeed affected the DSS in this overall population.

A similar phenomenon noted in the adult POIT subgroup reminded us that surveillance for stage I adult POIT in any grade should be applied cautiously. Our research showed that surveillance increased the risk of both recurrence and death in stage I adult POIT, although further assessment based on specific stages and grades was not applicable due to limited cases. A higher proportion of high grades may account for this, since more than half of the adult stage I POIT patients had G2-3 disease, and most of the recurrence or death occurred in G3 patients [10,11,20,23]. Older age, higher tumor grade, and incomplete staging were the identified risk factors for poor outcomes in previous studies [11,30]. In contrast, results in the pediatric subgroup in our meta-analysis revealed no statistical difference in recurrence or death in patients who chose surveillance or adjuvant chemotherapy, suggesting excellent outcomes in pediatric stage I POIT of any grade. This result was also in accordance with previous studies focusing on pediatric or adolescent POIT [8,9]. However, due to the limited numbers, the benefit of adjuvant chemotherapy in the pediatric subgroup remains unsolved. Based on these findings, extending the surveillance strategy for all ages in stage I POIT remains disputable.

Strangely, although adjuvant chemotherapy in stage I POIT significantly improved the DSS, it did not significantly decrease the incidence of recurrence. Wang et al. reported a 5-year disease-free survival rate of 91.7% and 96.0% (*p* = 0.46) in a large cohort of 75 patients with stage I POIT who underwent surveillance and adjuvant chemotherapy, respectively. Similar results were also found in research from Nasioudis et al. [24] (stage IA/IB G2-G3 POIT) and Bergamini et al. [11] (stage IA-IC G1-G3 POIT). However, since there was no RCT concerning surveillance or the adjuvant chemotherapy of POIT or MOGCTs, patients who would be administered surveillance or adjuvant chemotherapy were based on stages/grades as well as consultation between the physicians and patients. The non-randomized setting inevitably distributes most patients with POIT at higher risk of relapse to adjuvant chemotherapy, rather than surveillance, which artificially covers the true difference between two situations to a certain extent.

Our subsequent subgroup analysis further assessed the impact of adjuvant chemotherapy in possible circumstances of patients with POIT in each specific tumor stage and grade. However, no statistical difference in the incidence of recurrence or death was noted in the surveillance and adjuvant chemotherapy group among all the subgroups including stage IA G2-G3, stage I G3, stage IB-IC G1-G3, and stage IB-IC G2-G3 POIT. The inconsistent results between the overall cohort and these subgroup analyses could be explained by their inclusion of varied studies and diverse sample sizes. Nonetheless, the subgroup of POIT patients of G2-G3 or G3, irrespective of stage IA-IC, IB-IC, or IA, revealed a tendency for better survival outcomes in the forest plot, although it was not statistically significant. Therefore, tumor grade, rather than tumor stage, might have a more pronounced impact on the prognosis of stage I POIT [11]. Moreover, the synthetic results in our study differed from the findings reported in a single included study in some way, once again emphasizing the uncertain benefit and controversial surveillance in stage I POIT of all grades.

This study had several limitations. First, none of the included studies were a RCT and all were either prospective or retrospective studies, even though the heterogeneity was weak. Moreover, the 5-year RFS and OS/DSS rate were unavailable to synthesize due to the limited data and our strict inclusion criteria. In addition, the relatively small sample size of each tumor stage and grade increased the bias of the subgroup analysis, and some specific subgroups of the tumor grade or stage were not applicable.

## 5. Conclusions

Adjuvant chemotherapy significantly decreased the mortality rate in patients with stage I POIT and lowered the risk of recurrence in the adult subgroup. Surveillance administered in stage I POIT over IA G1 should be cautious, especially in adult patients.

## Figures and Tables

**Table 1:** The clinical characteristics of the included studies in our meta-analysis.

Author and Year Published	Design	Participant Disease	N (Stage I POIT)	Inclusion	Median Follow-Up *	Recurrence (Stage I POIT, Surveillance vs. Chemotherapy)	Death (Stage I POIT, Surveillance vs. Chemotherapy)
Bonazzi, 1994	RS	POIT	26	Pathology-confirmed diagnosis of POIT between 1982–1991, any stage	47 months	No event	No event
Mangili, 2010	RS	MOGCTs	28	MOGCT diagnosis between 1982–2008 with complete clinical data and outcomes, any stage	61 months	Two recurrences in the chemotherapy group	No event
Vicus, 2011	RS	POIT	32	POIT of any stage histologically diagnosed between 1970–2005	4.8 years	Two recurrences vs. one recurrence	Two deaths in the chemotherapy group
Alwazzan, 2015	RS	POIT	22	POIT of any stage/grade diagnosed between 1983 and 2013	60 months	No event	No event
Reddihalli, 2015	RS	POIT	16	POIT of any stage/grade diagnosed between 1999 and 2011 had the exact follow-up data and clinical outcomes	39 months	No event	No event
Mangili, 2017	RS	MOGCTs	49	Stage I MOGCTs diagnosed between 1982 and 2014 that had clear outcomes	59 months	Four cases vs. two cases	One death in surgery group
Newton, 2019	RS	MOGCTs	38	MOGCTs diagnosed between 2005 and 2016 of any stage/grade who had clear outcomes	56.6 months	Two recurrences in surgery group	No event
Pavone, 2020	RS	POIT	35	Pediatric (no more than 18 years old) POIT of any stage/grade	39.5 months	One relapse in surgery group (exclude IX G1)	No event
Wang, 2020	RS	POIT	75	Stage I POIT aged over 18 years who underwent fertility-sparing surgery between 1986 and 2018	80.2 months	Two recurrences in each group (5-year RFS of 91.7% vs. 96.0%)	One death in surgery group
Bergamini, 2020	RS	POIT	108	Post-puberal Stage I POIT diagnosed between 1985 and 2018 that had clear follow-up data	64.3 months	Nine cases vs. two cases	One death in surgery group
Mangili, 2021	PS	MOGCTs	23	Post-pubertal stage I MOGCT patients diagnosed between 2013 and 2019	46.2 months	No recurrence	No deaths
Nasioudis, 2021	RS	MOGCTs	272	IA/IB grade 2–3 POIT, yolk sac, or mixed MOGCTs diagnosed between 2004 and 2014 with at least 1 month of follow-up	63.8/61.7 months	NA	95.0% vs. 97.3% (5-year OS rate, *p* = 0.22)
Yuksel, 2021	RS	POIT	40	POIT patients aged between 15 and 39 years diagnosed between 1993 and 2019	60 months	No event	No event
Graham, 2022	RS	MOGCTs	39	Histological diagnosis of stage I MOGCTs between 2005 and 2016 that had clear outcomes	4.4 years	No event	No event
Zhang, 2022	RS	POIT	32	Histologically confirmed POIT of stage I (except IA G1) before 2016	24 months	One recurrence in each group	No event

Abbreviations: RS, retrospective study; PS, prospective study; MOGCTs, malignant ovarian germ cell tumors; POIT, pure ovarian immature teratoma; RFS, recurrence-free survival; OS, overall survival; MT, mature teratoma; NA, not applicable. * The median follow-up time was for the overall cohort in each study; 5-year RFS rate and 5-year OS rate for stage I POIT (except IA G1) were unable to be calculated in some studies.

## Data Availability

All data generated or analyzed during this study are included in this published article and its supplementary information files. The datasets used and/or analyzed during the current study are available from the corresponding author upon reasonable request.

## Supplementary Materials

The following supporting information can be downloaded at: https://www.mdpi.com/article/10.3390/cancers15061741/s1, Text S1: PRISMA checklist of this study (adapted from Ref. [31]); Text S2: The search strategy of our meta-analysis; Table S1: NOS for cohort studies included in this study; Table S2: The details of chemotherapy regimens and doses in the included studies; Figure S1: Impact of adjuvant chemotherapy on the recurrence and mortality in pediatric and adult POIT subgroups: (A) Recurrence in the pediatric subgroup, (B) mortality in the pediatric subgroup, (C) recurrence in the adult subgroup, and (D) mortality in the adult subgroup; Table S3: The detailed stages and grades of pediatric and adult subgroups in this study; Figure S2: Impact of adjuvant chemotherapy on the recurrence and mortality of the stage IA-IC G3 POIT subgroup: (A) recurrence, (B) mortality; Figure S3: Impact of adjuvant chemotherapy on the mortality in the stage IB-IC POIT subgroup (A) and IB-IC G2-G3 POIT subgroup (B).

## Abbreviations

POIT, pure ovarian immature teratoma; MOGCTs, malignant ovarian germ cell tumors; BEP, bleomycin, etoposide, and cisplatin; RFS, recurrence-free survival; DSS, disease-specific survival; NA, not applicable.
