# Supplementary material for: Role of Adjuvant Chemotherapy in Stage I Pure Ovarian Immature Teratoma: A Systematic Review and Meta-Analysis
Source: Cancers (Basel). 2023 Mar 13;15(6):1741. doi: 10.3390/cancers15061741 (PMC10046473; doi:10.3390/cancers15061741)
Supplement: Supplementary file 1 [file cancers-15-01741-s001.zip › cancers-2251901-supplementary.pdf]

Supplementary Materials: Role of Adjuvant Chemotherapy in Stage I Pure Ovarian Immature Teratoma: A Systematic Review and Meta-Analysis

Text S1. PRISMA Checklist.

Section and Topic	Item #	Checklist item	Location where item is reported
**TITLE**			
Title	1	Identify the report as a systematic review.	Page 1, line 1
**ABSTRACT**			
Abstract	2	See the PRISMA 2020 for Abstracts checklist.	Page 2
**INTRODUCTION**			
Rationale	3	Describe the rationale for the review in the context of existing knowledge.	Line 37 to 60
Objectives	4	Provide an explicit statement of the objective(s) or question(s) the review addresses.	Line 61 to 65
**METHODS**			
Eligibility criteria	5	Specify the inclusion and exclusion criteria for the review and how studies were grouped for the syntheses.	Line 78-91
Information sources	6	Specify all databases, registers, websites, organisations, reference lists and other sources searched or consulted to identify studies. Specify the date when each source was last searched or consulted.	Line 71-76
Search strategy	7	Present the full search strategies for all databases, registers and websites, including any filters and limits used.	Line 71-91
Selection process	8	Specify the methods used to decide whether a study met the inclusion criteria of the review, including how many reviewers screened each record and each report retrieved, whether they worked independently, and if applicable, details of automation tools used in the process.	Line 92-95
Data collection process	9	Specify the methods used to collect data from reports, including how many reviewers collected data from each report, whether they worked independently, any processes for obtaining or confirming data from study investigators, and if applicable, details of automation tools used in the process.	Line 98-105
Data items	10a	List and define all outcomes for which data were sought. Specify whether all results that were compatible with each outcome domain in each study were sought (e.g. for all measures, time points, analyses), and if not, the methods used to decide which results to collect.	Line 107 to 113
	10b	List and define all other variables for which data were sought (e.g. participant and intervention characteristics, funding sources). Describe any assumptions made about any missing or unclear information.	Line 98-104
Study risk of bias assessment	11	Specify the methods used to assess risk of bias in the included studies, including details of the tool(s) used, how many reviewers assessed each study and whether they worked independently, and if applicable, details of automation tools used in the process.	Line 103-105
Effect measures	12	Specify for each outcome the effect measure(s) (e.g. risk ratio, mean difference) used in the synthesis or presentation of results.	Line 115-125

Section and Topic	Item #	Checklist item	Location where item is reported
Synthesis methods	13a	Describe the processes used to decide which studies were eligible for each synthesis (e.g. tabulating the study intervention characteristics and comparing against the planned groups for each synthesis (item #5)).	Line 116-126
	13b	Describe any methods required to prepare the data for presentation or synthesis, such as handling of missing summary statistics, or data conversions.	Line 116-126
	13c	Describe any methods used to tabulate or visually display results of individual studies and syntheses.	Line 116-126
	13d	Describe any methods used to synthesize results and provide a rationale for the choice(s). If meta-analysis was performed, describe the model(s), method(s) to identify the presence and extent of statistical heterogeneity, and software package(s) used.	Line 116-126
	13e	Describe any methods used to explore possible causes of heterogeneity among study results (e.g. subgroup analysis, meta-regression).	Line 111-114
	13f	Describe any sensitivity analyses conducted to assess robustness of the synthesized results.	NA
Reporting bias assessment	14	Describe any methods used to assess risk of bias due to missing results in a synthesis (arising from reporting biases).	Line 125-126
Certainty assessment	15	Describe any methods used to assess certainty (or confidence) in the body of evidence for an outcome.	Line 120-123
**RESULTS**			
Study selection	16a	Describe the results of the search and selection process, from the number of records identified in the search to the number of studies included in the review, ideally using a flow diagram.	Line 130-134
	16b	Cite studies that might appear to meet the inclusion criteria, but which were excluded, and explain why they were excluded.	NA
Study characteristics	17	Cite each included study and present its characteristics.	Line 132-134
Risk of bias in studies	18	Present assessments of risk of bias for each included study.	Line 138-139
Results of individual studies	19	For all outcomes, present, for each study: (a) summary statistics for each group (where appropriate) and (b) an effect estimate and its precision (e.g. confidence/credible interval), ideally using structured tables or plots.	Line 142-150
Results of syntheses	20a	For each synthesis, briefly summarise the characteristics and risk of bias among contributing studies.	Line 156-184
	20b	Present results of all statistical syntheses conducted. If meta-analysis was done, present for each the summary estimate and its precision (e.g. confidence/credible interval) and measures of statistical heterogeneity. If comparing groups, describe the direction of the effect.	Line 156-184
	20c	Present results of all investigations of possible causes of heterogeneity among study results.	Line 156-184
	20d	Present results of all sensitivity analyses conducted to assess the robustness of the synthesized results.	NA
Reporting biases	21	Present assessments of risk of bias due to missing results (arising from reporting biases) for each synthesis assessed.	Line 156 -184
Certainty of evidence	22	Present assessments of certainty (or confidence) in the body of evidence for each outcome assessed.	Line 142-184
**DISCUSSION**			

Section and Topic	Item #	Checklist item	Location where item is reported
Discussion	23a	Provide a general interpretation of the results in the context of other evidence.	Line 187-217
	23b	Discuss any limitations of the evidence included in the review.	Line 254-259
	23c	Discuss any limitations of the review processes used.	Line 254-259
	23d	Discuss implications of the results for practice, policy, and future research.	Line 218-253
**OTHER INFORMATION**			
Registration and protocol	24a	Provide registration information for the review, including register name and registration number, or state that the review was not registered.	Line 68-69
	24b	Indicate where the review protocol can be accessed, or state that a protocol was not prepared.	Line 68-69
	24c	Describe and explain any amendments to information provided at registration or in the protocol.	NA
Support	25	Describe sources of financial or non-financial support for the review, and the role of the funders or sponsors in the review.	Line 283 to 286
Competing interests	26	Declare any competing interests of review authors.	Line 280-281
Availability of data, code and other materials	27	Report which of the following are publicly available and where they can be found: template data collection forms; data extracted from included studies; data used for all analyses; analytic code; any other materials used in the review.	Line 404 to 415

From: Page MJ, McKenzie JE, Bossuyt PM, Boutron I, Hoffmann TC, Mulrow CD, et al. The PRISMA 2020 statement: an updated guideline for reporting systematic reviews. BMJ 2021;372:n71. doi: 10.1136/bmj.n71.

Text S2. Search strategy.

Pubmed search strategy. Publication date to December 15, 2022.

Search Number	Query	Results
1	"ovarian immature teratoma" OR "ovarian malignant teratoma" OR ("immature teratoma" AND "ovary") OR ("malignant teratoma" AND "ovary") OR ("immature teratoma" AND ovar*) OR (IMT)	11,831
2	"Malignant ovarian germ cell tumors" OR "Malignant ovarian germ cell tumor" OR MOGCT OR (GCT AND ovar*)	791
3	"Teratoma"[Mesh] AND "immature"	1,187
4	"Chemotherapy, Adjuvant"[Mesh] OR "Drug Therapy, Adjuvant" OR "Adjuvant Chemotherapy" OR "Adjuvant Drug Therapy"	64,192
5	#1 OR #2 OR #3	13,362
6	#4 AND #5	237
7	"1970/01/01"[Date - Publication] : "2022/12/13"[Date - Publication]	31,705,917
8	#6 AND #7	237

Search detail: ("ovarian immature teratoma"[All Fields] OR "ovarian malignant teratoma"[All Fields] OR ("immature teratoma"[All Fields] AND "ovary"[All Fields]) OR ("malignant teratoma"[All Fields] AND "ovary"[All Fields]) OR ("immature teratoma"[All Fields] AND "ovar*"[All Fields]) OR "IMT"[All Fields] OR ("Malignant ovarian germ cell tumors"[All Fields] OR "Malignant ovarian germ cell tumor"[All Fields] OR ("mogct"[All Fields] OR "mogcts"[All Fields]) OR (("gene

cell tissue"[Journal] OR "gct"[All Fields]) AND "ovar*"[All Fields])) OR ("Teratoma"[MeSH Terms] AND "immature"[All Fields])) AND ("chemotherapy, adjuvant"[MeSH Terms] OR "drug therapy adjuvant"[All Fields] OR "Adjuvant Chemotherapy"[All Fields] OR "Adjuvant Drug Therapy"[All Fields]) AND 1970/01/01:2022/12/13[Date - Publication].

EMBASE. Publication date to December 15, 2022. Database: Embase 1970 to December 15, 2022.

#	Searches	Results
1	'ovarian immature teratoma'/exp OR 'ovarian immature teratoma' OR 'ovarian malignant teratoma'/exp OR 'ovarian malignant teratoma' OR (('immature teratoma'/exp OR 'immature teratoma') AND ('ovary'/exp OR 'ovary')) OR (('malignant teratoma'/exp OR 'malignant teratoma') AND ('ovary'/exp OR 'ovary')) OR (('immature teratoma'/exp OR 'immature teratoma') AND ovar*) OR imt	21589
2	'malignant ovarian germ cell tumors' OR 'malignant ovarian germ cell tumor'/exp OR 'malignant ovarian germ cell tumor' OR 'mogct'/exp OR mogct OR (gct AND ovar*)	5454
3	"Teratoma" AND "immature"	2698
4	'drug therapy, adjuvant' OR 'adjuvant chemotherapy' OR 'adjuvant drug therapy'	86541
5	#1 OR #2 OR #3	28370
6	#4 AND #5	491
7	#6 AND [01-01-1970]/sd NOT [14-12-2022]/sd	491

Cochrane. Database: Cochrane Central Register of Controlled Trials（CENTRAL）and Cochrane Database of Systematic Reviews to December 15, 2022.

#	Searches	Results
1	"ovarian immature teratoma" OR "ovarian malignant teratoma" OR "immature teratoma" AND "ovary" OR ("malignant teratoma" AND "ovary") AND ("immature teratoma" AND ovar*) OR (IMT)	24 (6 reviews + 2 protocols + 16 trials)
2	"Malignant ovarian germ cell tumors" OR "Malignant ovarian germ cell tumor" OR MOGCT OR (GCT AND ovar*)	22 (3 reviews + 19 trials)
3	"Teratoma" AND "immature"	10 (1 review + 9 trials)
4	MeSH descriptor: [Chemotherapy, Adjuvant] explode all trees	4140
5	#1 OR #2 OR #3	39
6	#5 AND #6	3
7	#5 AND #6 with Cochrane Library publication date Between Jan 1970 and Dec 2022	3

Web of Science. Database: WOS, MEDLINE, RSCI. Language: Auto. Time range: 1970 to 2022.

#	Searches	Results
1	TS = (ovarian malignant teratoma) OR (immature teratoma AND ovary) OR (malignant teratoma AND ovary) OR (immature teratoma AND ovar*) OR IMT OR (ovarian immature teratoma)	12202
2	TS = (Malignant ovarian germ cell tumors) OR (Malignant ovarian germ cell tumor) OR MOGCT OR (GCT AND ovar*)	1372
3	TS = (teratoma) AND (immature)	1507
4	#3 OR #2 OR #1	13934
5	TS = (Chemotherapy, Adjuvant) OR (Drug Therapy, Adjuvant) OR (Adjuvant Chemotherapy) OR (Adjuvant Drug Therapy)	74206
6	#4 AND #5	312

ClinicalTrials.Gov. **Condition or disease:** ovarian immature teratoma OR ovarian malignant teratoma OR early-stage ovarian immature teratoma OR Malignant ovarian

germ cell tumors OR MOGCT OR IT. **Other items:** Adjuvant Chemotherapy OR Postoperative chemotherapy OR Chemotherapy. Items: 14.

Table S1. Risk of bias cohort studies using Newcastle-Ottawa Quality Assessment Scale.

	**Selection**				**Comparability**	**Outcome**			
**Study**	Representativeness of the exposed cohort	Selection of the non-exposed cohort	Ascertainment of exposure	Demonstration that outcome of interest was not present at start of study	Comparability of cases and controls	Assessment of outcome	follow-up long enough for outcomes	Adequacy of follow up	**Total**
**Bonazzi 1994**	0	1	1	1	1	1	1	1	7
Mangili 2010	1	1	1	1	1	1	1	1	8
Vicus 2011	0	1	1	1	1	1	1	1	7
Alwazzan 2015	0	1	1	1	1	1	1	1	7
Reddihalli 2015	1	1	1	1	1	1	1	1	8
Mangili 2017	1	1	1	1	1	1	1	1	8
Newton 2019	1	1	1	1	1	1	1	1	8
Pavone 2019	1	1	1	1	1	1	1	1	8
Wang 2020	1	1	1	1	1	1	1	1	8
Bergamini 2020	1	1	1	1	2	1	1	1	9
Mangili 2021	1	1	1	1	1	1	1	1	8
Nasioudis 2021	1	1	1	1	1	0	1	1	7
Yuksel 2021	1	1	1	1	1	1	1	1	8
Graham 2022	1	1	1	1	1	1	1	1	8
Zhang 2022	1	1	1	1	1	1	1	1	8

Table S2. The details of chemotherapy regimens and dose in included studies.

**Author-publish year**	**Participants disease**	**Chemotherapy regimens**	**Dose of drugs**
Bonazzi 1994	POIT	BVP every 3 weeks for 3-4 courses (before 1989); then BEP every 8 days for 3-4 course	BVP: B 30mg, D1/8/15; V 0.15mg/kg, D1/2; P 20mg/m^2 D1-5<br>BEP: B 30mg D1; E 75mg/m^2 D1/2
Mangili 2010	MOGCTs	BEP every 3 weeks for 3-4 courses	Unspecified
Vicus 2011	POIT	BEP (7 cases), EP (6 cases), VAC (1 case)	Unspecified

Alwazzan 2015	POIT	VAC (before 1994, 11 cases) twelve cycles every 21 days; EP (10 cases) four cycles every 21 days	VAC: V 1.5 mg/m² D1/15; A 350mg/m² D1-5; C 150mg/m² D1-5<br>EP: E 100mg/m² D1-5; P 20mg/m² D1-5
Reddihalli 2015	POIT	BEP 3-4 cycles every 3 weeks	Unspecified
Mangili 2017	MOGCTs	Unspecified	Unspecified
Newton 2019	MOGCTs	BEP every 3 weeks (most cases); JEB every 3 weeks	Unspecified
Pavone 2020	POIT	BEP or BVP every 3 weeks	Unspecified
Wang 2020	POIT	Most cases were BEP or BVP every 3 weeks	Unspecified
Bergamini 2020	POIT	BEP every 3 weeks for 3 to 5 cycles or POMB/ACE every 3 weeks for 10 to 12 weeks	Unspecified
Mangili 2021	MOGCTs	BEP every 3 weeks	Unspecified
Nasioudis 2021	MOGCTs	Unspecified	Unspecified
Yuksel 2021	POIT	BVP, BEP, or EP	Unspecified
Graham 2022	MOGCTs	BEP	Unspecified
Zhang 2022	POIT	BEP or BVP every 3 weeks for 3-4 courses	BEP: B 20mg/m² D2; E 100mg/m² D1-3; P 100mg/m² divided in D1-3;<br>BVP: B 20mg/m² D2; V 1-1.5mg/m² D1-2; P 100mg/m² divided in D1-3

Abbreviations: POIT, pure ovarian immature teratoma; MOGCTs, malignant ovarian germ cell tumors; BEP, bleomycin, etoposide, and cisplatin; BVP, bleomycin, vincristine, and cisplatin; EP, Etoposide and Cisplatin; VAC, Vincristine, Actinomycin D, and Cyclophosphamide; JEB, bleomycin, etoposide, and carboplatin; POMB/ACE, cisplatin, vincristine, methotrexate, bleomycin, actinomycin, cyclophosphamide, and etoposide.

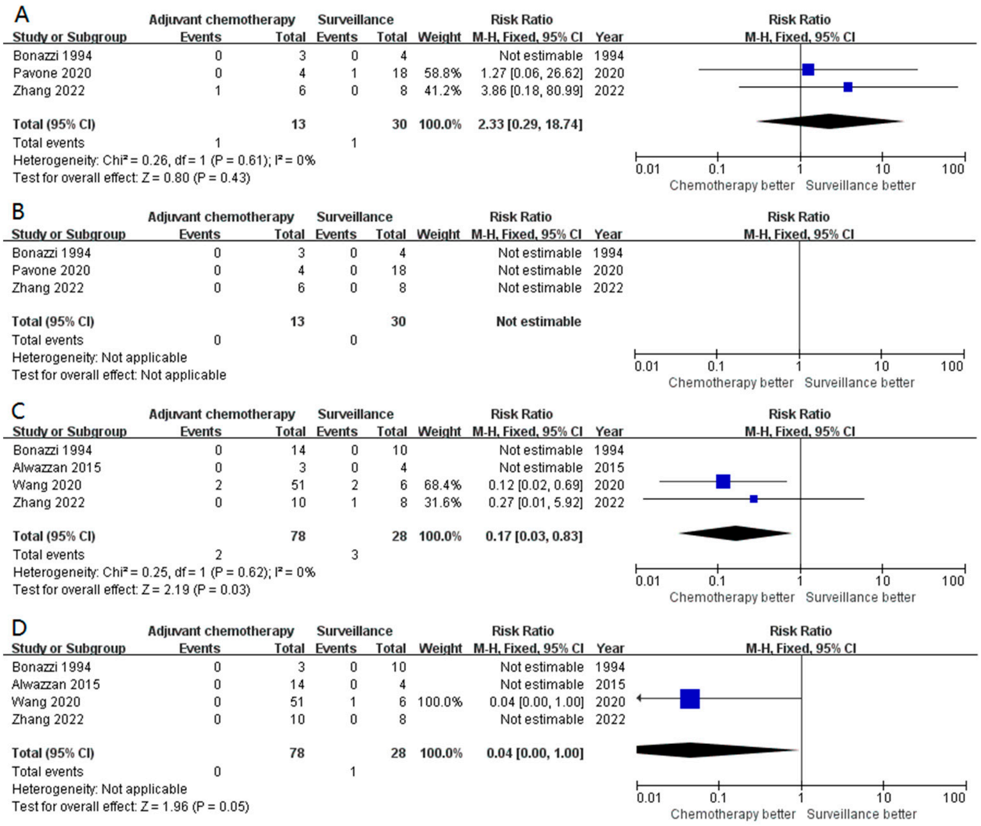

Figure S1. Survival outcomes in pediatric and adult subgroup.

Table S3. The detailed stage and grade for pediatric and adult subgroups in this study.

Subgroup	Stage	Cases	Grade	Cases
Pediatric subgroup	IA	16	G2	11
Total N = 43			G3	5
	IX	1	G3	1
	IC	26	G1	9
			G2	7
			G3	10
Adult subgroup	IA	29	G2	10
Total N = 106			G3	9
			G2 or G3	10
	IB	1	G2	1
	IC	41	G1	11
			G2	7
			G3	11
			G1 - G3 (unspecific)	12
	IX	35	G2 or G3	35

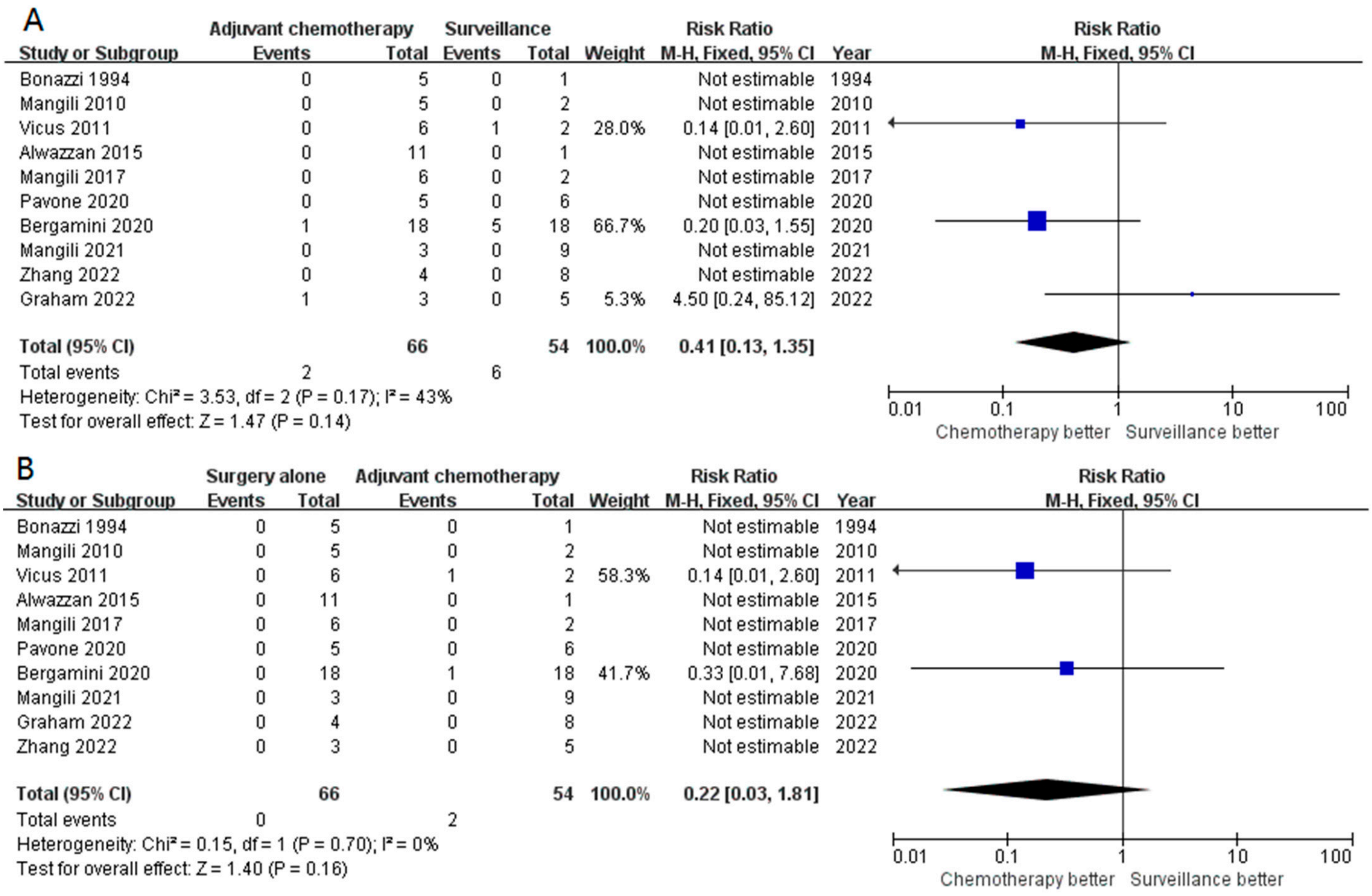

Figure S2. Recurrence and death in I G3 subgroup.

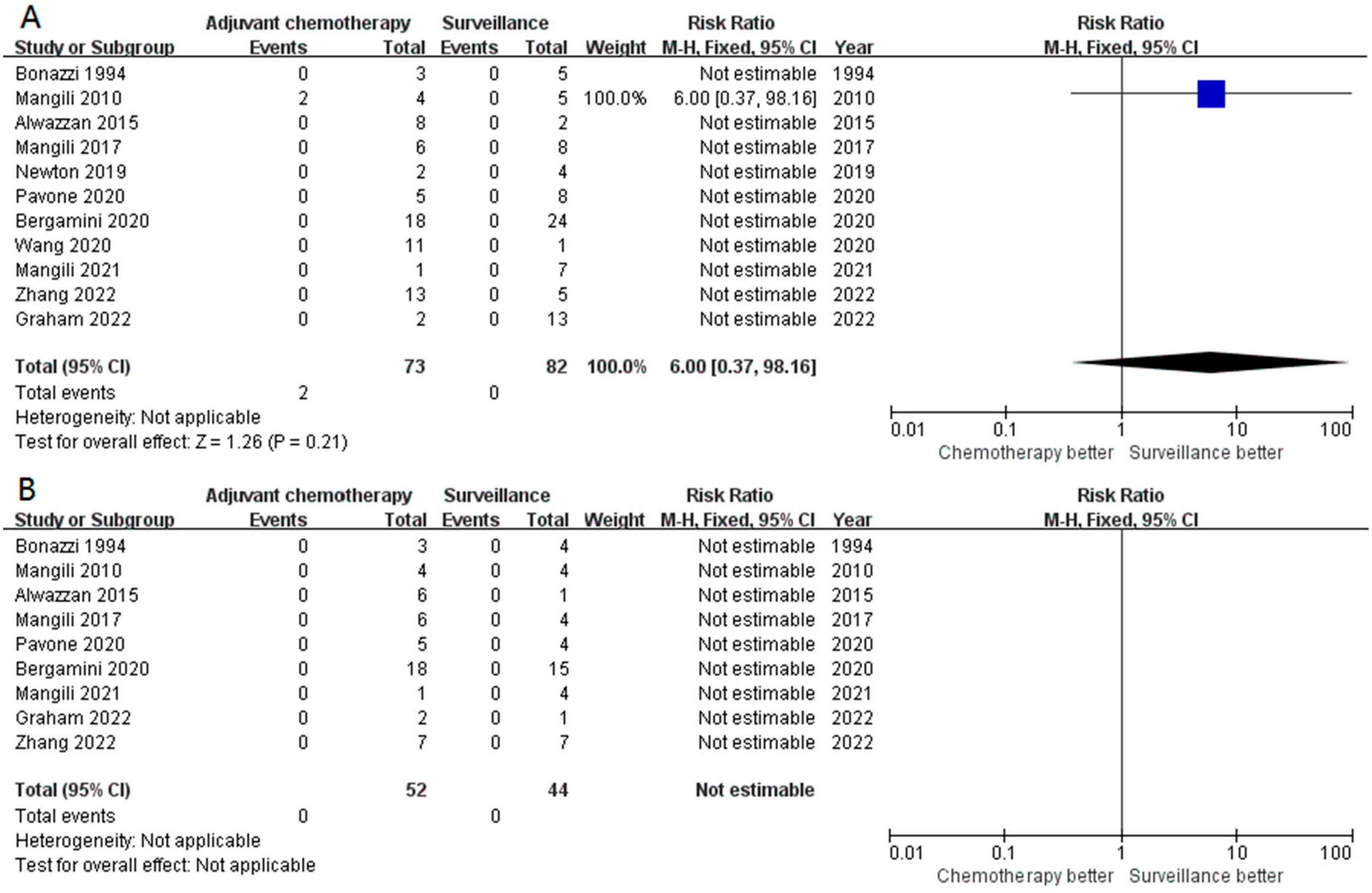

Figure S3. Death in IB-IC and I B G2-3 subgroup.
